# AOAH remodels arachidonic acid-containing phospholipid pools in a model of interstitial cystitis pain: A MAPP Network study

**DOI:** 10.1371/journal.pone.0235384

**Published:** 2020-09-14

**Authors:** Wenbin Yang, Ryan E. Yaggie, Anthony J. Schaeffer, David J. Klumpp

**Affiliations:** 1 Department of Urology, Feinberg School of Medicine, Northwestern University, Chicago, IL, United States of America; 2 Department of Microbiology-Immunology, Feinberg School of Medicine, Northwestern University, Chicago, IL, United States of America; University of Arizona College of Medicine, UNITED STATES

## Abstract

Interstitial cystitis/bladder pain syndrome (IC) is a debilitating condition of chronic pelvic pain with unknown etiology. Recently, we used a genetic approach in a murine model of IC to identify the lipase acyloxyacyl hydrolase (AOAH) as a modulator of pelvic pain. We found that AOAH-deficient mice have elevated pelvic pain responses, and AOAH immunoreactivity was detected along the bladder-brain axis. Lipidomic analyses identified arachidonic acid (AA) and its metabolite PGE_2_ as significantly elevated in the sacral spinal cord of AOAH-deficient mice, suggesting AA is a substrate for AOAH. Here, we quantified the effects of AOAH on phospholipids containing AA. Spinal cord lipidomics revealed increased AA-containing phosphatidylcholine in AOAH-deficient mice and concomitantly decreased AA-phosphatidylethanolamine, consistent with decreased CoA-independent transferase activity (CoIT). Overexpression of AOAH in cell cultures similarly altered distribution of AA in phospholipid pools, promoted AA incorporation, and resulted in decreased membrane fluidity. Finally, administration of a PGE_2_ receptor antagonist reduced pelvic pain in AOAH-deficient mice. Together, these findings suggest that AOAH represents a potential CoA-independent AA transferase that modulates CNS pain pathways at the level of phospholipid metabolism.

## Introduction

Interstitial cystitis/bladder pain syndrome (IC) is a chronic bladder syndrome associated with severe pelvic pain and voiding dysfunction, causing untold suffering in as many as many as eight million patients in the United States, with women comprising ~90% of patients [1]. IC remains a clinical challenge as the etiology is unknown, and no effective therapy exists. Because of the profound impact of chronic pelvic pain, the National Institute of Diabetes and Digestive and Kidney Diseases established the Multi-Disciplinary Approaches to Chronic Pelvic Pain Research Network, its flagship effort to understand mechanisms underlying urologic chronic pelvic pain syndromes [2, 3]. As part of these studies, we recently employed a murine neurogenic cystitis model that recapitulates key aspects of IC [4] to identify loci that modulate pelvic pain using a quantitative trait loci genetic strategy, and we found that acyloxyacyl hydrolase (AOAH) is a novel modulator of pelvic pain [5]. AOAH-deficient mice were found to have spontaneously elevated mechanical allodynia of the pelvic region relative to wild type mice, an evoked behavioral response consistent with pelvic pain. AOAH-deficient mice also exhibited increased pelvic allodynia in a bladder infection model of IC and a neurogenic cystitis model of IC. Similarly, AOAH-deficient mice had elevated visceromotor reflex response to bladder distension. These findings were accompanied by increased markers of mast cell activation, AOAH staining was detected at sites along the micturition pathway including the bladder, the dorsal horn of the sacral spinal cord, and the Barrington’s nucleus. Finally, AOAH-deficient mice had elevated levels of vascular endothelial growth factor in the bladder, an emerging biomarker for chronic pelvic pain.

AOAH is best known as a neutrophil lipase that detoxifies bacterial lipopolysaccharide (LPS) by cleaving secondary acyl chains from the lipid A moiety [6]. In addition to detoxification of LPS, AOAH has been shown to mediate transfer of LPS acyl chains to acceptor lipids and act upon glycero- and phospholipids, suggesting that AOAH biochemical functions may impact diverse physiologic processes [6]. Indeed, genetic studies have implicated *Aoah* polymorphisms in cardiovascular disease, body mass, and chronic inflammation [7–9]. Increased *Aoah* expression has even been detected in bladder biopsies from IC patients [10]. To understand the role of AOAH lipase activity in pelvic pain, we recently examined lipids of the sacral spinal cord and found that AOAH-deficient mice have elevated concentrations of arachidonic acid (AA) and prostaglandin E2 (PGE_2_) [11]. These findings are consistent with many clinical studies linking pain to AA and its eicosanoid metabolites including in neuropathic pain and osteoarthritis pain [12, 13]. Moreover, the finding of elevated AA in AOAH-deficient mice suggests that AOAH plays a role in AA homeostasis.

AA and its esterified arachidonates are ubiquitous components of membrane phospholipids. AA is an n-6 polyunsaturated fatty acid that is the precursor of bioactive lipid mediators such as prostaglandins, leukotrienes, thromboxanes, and P450-derived metabolites [14]. The incorporation and distribution of AA among phospholipids occurs through the Lands cycle, a remodeling process that includes a deacylation-reacylation cycle of membrane phospholipids [15]. Three distinct reactions have been proposed for reincorporation of AA to lysophospholipids: (i) acyl-CoA-dependent, (ii) CoA-dependent, and (iii) CoA-independent reactions [16]. However, despite having been characterized enzymatically in cellular extracts and membrane fraction for decades, the precise identity of the protein mediating CoIT remodeling of AA phospholipid pools remains unknown [16]. Here, we examined the effects of AOAH on phospholipid pools in vivo and in vitro. We found that AOAH mediates remodeling of phospholipid pools consistent with arachidonyl CoA-independent activity and that targeting PGE_2_ signaling reduces pain in AOAH-deficient mice. Thus, these studies describe a novel level of modulation of pelvic pain mediated by AOAH.

## Materials and methods

### Animals

10–12 week old female wild-type C57BL/6J mice were purchased from Jackson laboratory (Bar Harbor, ME). AOAH knockout mice (*Aoah*^*-/-*^) on the C57BL/6 background, a gift from Dr. Robert Munford (National Institute of Allergy and Infectious Diseases, Bethesda, MD), were generated as described previously [17]. All experiments were performed using protocols approved by Northwestern University Institutional Animal Care and Use Committee. All mice were housed under specific pathogen-free conditions in the barrier facilities of the Center for Comparative Medicine and maintained on a regular 12:12-h light dark cycle with food and water ad libidum. Spinal cord segments were dissected from euthanized mice relative to anatomical landmarks identify S1-S3 [18]. Briefly, the ligamentum flavum was cut with a small scissor to expose the sacral spinal cord with a dorsal artery in the middle spinal column. The sacral cord was excised and placed immediately into ice-cold saline, and muscle and other soft tissues on the column were removed prior to sample preparation. The small size of the excised tissue precluded sub-dissection.

### Intrathecal injection

EP1 antagonist ONO-8711 (Cayman chemical, Ann Arbor, MI) was dissolved in DMSO to 20mg/ml as a stock and diluted in PBS to 10 nM for use. Drug administration was performed in a volume of 5 μl by a 30-gauge needle connected to a 25 μl Hamilton syringe (Reno, NV) through an intervertebral space between L5 and L6, as described previously [19]. Successful intrathecal (i.t.) injection was verified by a lateral tail-flick. Mice were administered with 5 μl saline or ONO-8711, 1 h before allodynia testing.

### Allodynia testing

Pelvic hyperalgesia and mechanical allodynia were quantified using von Frey filaments (Stoelting, Kiel, WI) applied to the abdomen as described previously [20]. Mice were tested in individual Plexiglas chambers with a stainless steel wire grid floor (mouse acclimation period of 5–10 min before testing). Frequency of withdrawal responses to the application of von Frey filaments to the abdomen was tested using five individual fibers with forces of 0.04, 0.16, 0.4, 1, and 4 g. Each filament was applied for 1 s for a total 10 times, and the filaments were tested in ascending order of force. Stimulation was confined to the lower abdominal area in the general vicinity of the bladder. Three types of behaviors were considered as positive responses to pelvic stimulation: 1) sharp retraction of the abdomen; 2) immediate licking or scratching of the area of filament stimulation; or 3) jumping. At each force, data were expressed as the frequency of response (i.e., for each fiber, the mean number of responses from 10 applications of the fiber, expressed as a percentage). Alternatively, for quantifiying the effect of EP1 antagonism following intrathecal drug administration, baseline responsiveness was quantified prior to drug or vehicle administration to determine the frequency of responses to all five fibers (i.e., number of responses to 50 combined stimulations); 60 min. after drug administration, mice were retested with five fibers to determine the change in aggregate response relative to baseline responses and expressed as percent change.

### Cell culture

Embryonic mouse hypothalamus cell line N42 and human embryonic kidney cell line 293T were maintained in DMEM medium supplemented with 10% (v/v) fetal bovine serum and 100U/ml penicillin and streptomycin.

### AOAH-deficient N42 cell line

N42 cells were transfected with sgRNA (5’-AAGGGTATTGATCCGAAAGA-3’) specific for mouse chromosome 13, nucleotides 20,914,951–20,914,968 (NCBI CCDS26266.1) and Cas9 constructs using Genome-CRISP CRISPR-Cas9 kit (GeneCopoeia, Rockville, MD) and selected for hygromycin resistance. Single-cell cultures were expanded from 96-well plates and sequenced to verify homozygous mutation, and clone N42-672 was used as an AOAH-deficient cell line for subsequent studies.

### Recombinant pLenti-LacZ and pLenti-AOAH3 virus

*LacZ* and a cDNA encoding human AOAH3 were recombined into the *attL* sites of pENTR/SD-TOPO Using LRClonase II (ThermoFisher, Waltham, MA). pENTR-LacZ and pENTR-AOAH3 were shuttled into pLenti6.3/V5-Dest (ThermoFisher), and constructs were verified by sequencing. HEK-293FT cells were transfected with pLenti constructs and Virapower Lentiviral support kit (ThermoFisher) to produce lentivirus. AOAH-deficient N42-672 cells were transduced with either recombinant lentivirus to make N42 lenti-hAOAH3 and N42 lenti-lacZ cell lines and similarly transduced 293 cell lines.

### Lipid extraction

The total lipids of phosphatidylcholine (PC) and phosphatidylethanolamine (PE) were extracted by the Bligh and Dyer method [21], after spiking the samples with 200ng each of PC 17:0 and PE 17:0 as internal standards (Cayman). Tissue samples or cell pellets were immediately frozen, weighted and kept at -80°C.

### PC and PE analysis by shotgun lipidomics

Lipid extracts were analyzed on a hybrid triple/quadrupole linear ion trap mass spectrometer (QTRAP 6500, AB Sciex, Framingham, MA), using direct flow infusion. Molecular lipids were analyzed in both positive and negative ion modes using multiple precursor ion scanning-based methods. The molecular species of PC and PE were identified and quantified by normalizing to respective synthetic C_17_ internal standards. Data processing was performed with LipidView (AB Sciex).

### Molecular analysis of PC and PE species by UHPLC-MS

Molecular species of various lipids were analyzed by UHPLC-MS using a mass spectrometer (QTRAP 6500) coupled with an Agilent 2600 UHPLC system. The samples were diluted with Solvent A (80/19.5/0.5 chloroform/methanol/water). A normal phase silica column (15 cm × 2.1 mm, Phenomemex, Torrance, CA) was used for LC separation. Mobile phase B consisted of 60/34.5/5.5 chloroform/methanol/water (v/v). Both A and B were supplemented with 0.2% of ammonium acetate. Chromatographic separations were accomplished with 100% solvent A for 5 min, then a linear gradient of 100% solvent A to 100% solvent B from 5 to 30 min, and 100% solvent B from 30 to 35 min. The column temperature was held at 25°C, and the flow rate was set to 350 μl min^−1^. Electrospray ionization (ESI)-MS was performed in both positive and negative multiple reaction monitoring (MRM) mode for the quantitative and qualitative analysis of PC and PE species (by comparison with a previously published database) [22–24]. The spray voltage was 4.5 kV, and the source temperature was set at 450°C. Mass spectra were acquired and recorded by Analyst software (AB Sciex). The position of the acyl groups in each PC and PE (conventionally sn-1 acyl group, followed by sn-2 acyl group) was assumed based on the known preponderance of saturated acyl groups at the sn-1 position and unsaturated acyl groups at sn-2. Quantitation of individual molecular species of PC and PE was performed from the relative peak area of the various species and the corresponding internal standards (PC 17:0 and PE 17:0). Data processing was performed by Multiquant (AB Sciex).

### Measurement of [^2^H]AA incorporation into cellular lipids

Cells were incubated with exogenous deuterated 1μM [^2^H]AA (d8-AA, Cayman Chemical) for 30 min, then the reaction was stopped by replacing the incubation medium with ice-cold 0.1% Triton X-100, and total lipids were extracted. The various [^2^H]AA -containing phospholipids were analyzed by LC-MS as described above.

### Fluorescence recovery after photobleaching (FRAP) experiments

Cells were incubated for approximately 30 min with lipophilic dye BODIPY FL C12 (Molecular Probes, Eugene, OR). Fluorescence microscopy was carried out using a Nikon X1 spin flask confocal microscope (Nikon), and images were acquired using a digital black and white camera (Hamamatsu, Bridgewater, NJ) and NIS-Elements software (Nikon, Melville, NY). Cells were cultured in a chambered coverslip and placed on a temperature-controlled stage under the microscope objective lens. A 60x oil immersion lens was used. The confocal spot was scanned for about 0.5s in the middle of the cell to create a circular bleach zone. The confocal spot was then scanned to record a sequence of images of the cell at 0.5-s intervals. Images were analyzed using ImageJ with the simFRAP plugin [25].

### Statistical analysis

Results were expressed as mean± SEM and analyzed for statistical significance by t-test (two groups) or by the ANOVA (more than two groups), followed by a post hoc test comparison with Dunnett’s multiple comparison, using GraphPad Prism (San Diego, CA). A value of P < 0.05 was considered statistically significant.

## Results

### Characterization of sacral spinal cord phospholipids

**Fig 1:**
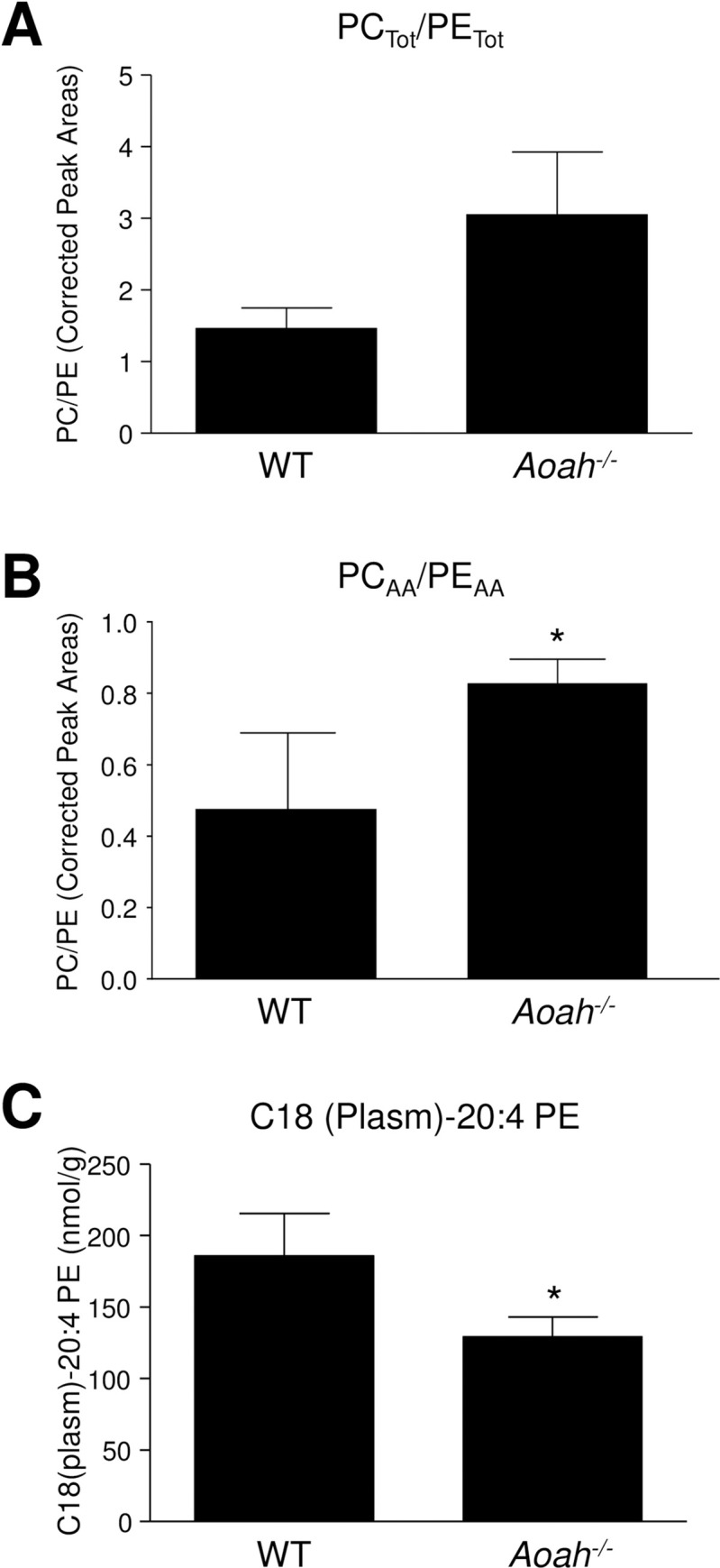
Diminished AA incorporation into phosphatidyl ethanolamine in AOAH-deficient mice. Shotgun lipidomics was performed on sacral spinals cords of female C57BL6/J mice or AOAH-deficient mice. ***A*,** The overall ratio of PC-to-PE across all detected species (i.e., different acyl chains) was not significantly different between wild type and AOAH-deficient mice. ***B***, In PC and PE with AA in the sn2 position (PC_AA_ and PE_AA_, respectively), significantly less AA accumulated in PE, relative to PC, resulting in greater PC_AA_/PE_AA_ ratios for spinal cords of AOAH-deficient mice relative to wild type spinal cords (n = 3–5 mice, *P<0.05,unpaired *t* test). ***C***, AOAH-deficient spinal cords contained significantly less C18 (Plasm)-20:4 PE than wild type (n = 3–5 mice, *P < 0.05, Mann-Whitney).

Since we recently found increased free AA in spinal cords of female AOAH-deficient mice [11], we examined phospholipid pools of AA female mice. We used an untargeted shotgun lipidomics strategy consisting of the ordered acquisition of mass ion trap detection to investigate differences in phosphatidyl choline and phosphatiyl ethanolamine (PC and PE, respectively) under positive and negative ionization mode of lipids extracted from spinal cord segments S1-S3. No significant difference was observed between wild type and AOAH-deficient mice in the overall ratio of PC/PE (Fig 1A). However, the ratio of AA-containing PC/PE was significantly different (Fig 1B), where the sacral spinal cords of AOAH-deficient mice exhibited significantly higher PC_AA_/PE_AA_ than wild type mice. To confirm the findings of shotgun lipidomics, ESI-MS was performed in positive MRM mode for the quantitative analysis of a specific AA-containing PE, C18 (Plasm)-20:4 PE (Fig 1C). Consistent with the findings of shotgun lipidomics, wild type mice contained more C18 (Plasm)-20:4 PE than AOAH-deficient mice. Together, these data suggest that AOAH mediates sequestration of AA in specific PE species in the CNS.

### AOAH alters AA distribution among phospholipids in vitro

**Fig 2:**
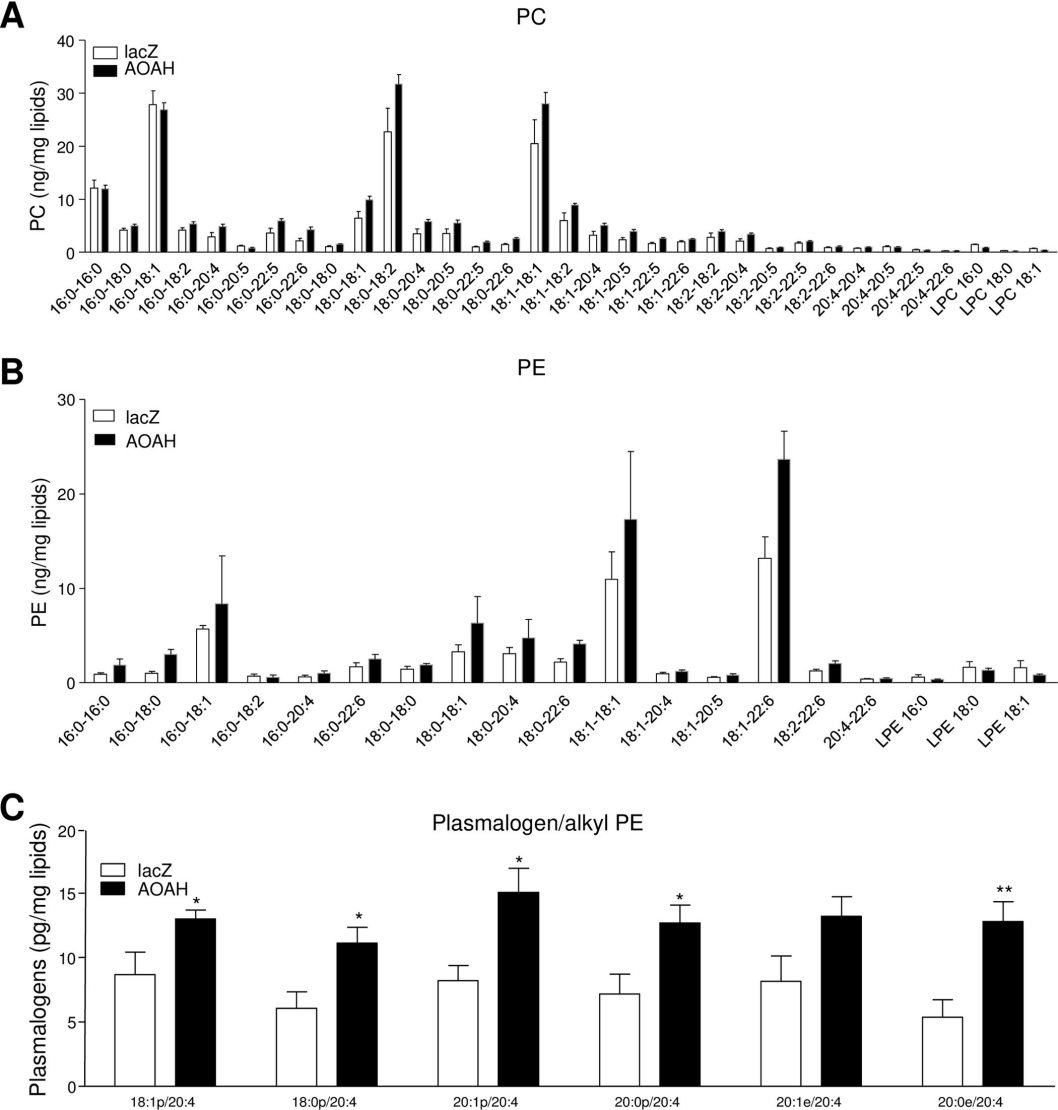
AOAH promotes AA accumulation in plasmalogen/alkyl PEs. Analysis of PC and PE species in 293T cells. Compared to 293T lenti-LacZ cells (open bars), 293T lenti-hAOAH3 cells (filled bars) did not modify the level of AA esterified in di-acyl PC (A) and PE (B), but AOAH increased the level of AA in plasmalogen or alkyl PE (C) (n = 5, *P < 0.05, **P < 0.01, 2-way ANOVA).

Although lipidomics showed that sacral spinal cords of female wild type mice contained more AA-containing PE than AOAH-deficient mice, it was unclear whether AOAH could directly modulate the sequestration of AA among CNS phospholipids. Microarray analyses showed that variant 3 of human AOAH (hAOAH3) was highly expressed in the CNS compared to other hAOAH variants [5, 11, 26]. We generated a recombinant lentivirus encoding hAOAH3 and, after confirming that HEK293 cells lack AOAH expression by immunoblotting (not shown), transduced HEK293 cells with AOAH3 or lacZ to obtain the 293 lenti-hAOAH3 and 293 lenti-lacZ cells, respectively. The molecular species of PC and PE, along with lysoPC (LPC) and lysoPE (LPE), were analyzed by UHPLC-MS. No significant differences in PC or PE species were observed between lacZ and AOAH 293 lines (Fig 2A and 2B, respectively). However, when AA-containing plasmalogens and alkyl PE were profiled, several species were significantly increased in cells expressing AOAH (Fig 2C). Thus, although AOAH did not appear to modify levels of AA esterified in di-acyl PC and PE, AOAH increased accumulation of AA in both plasmalogen and alkyl PEs.

### Metabolic analysis of exogenous AA incorporation into PC and PE of N42 cells

**Fig 3:**
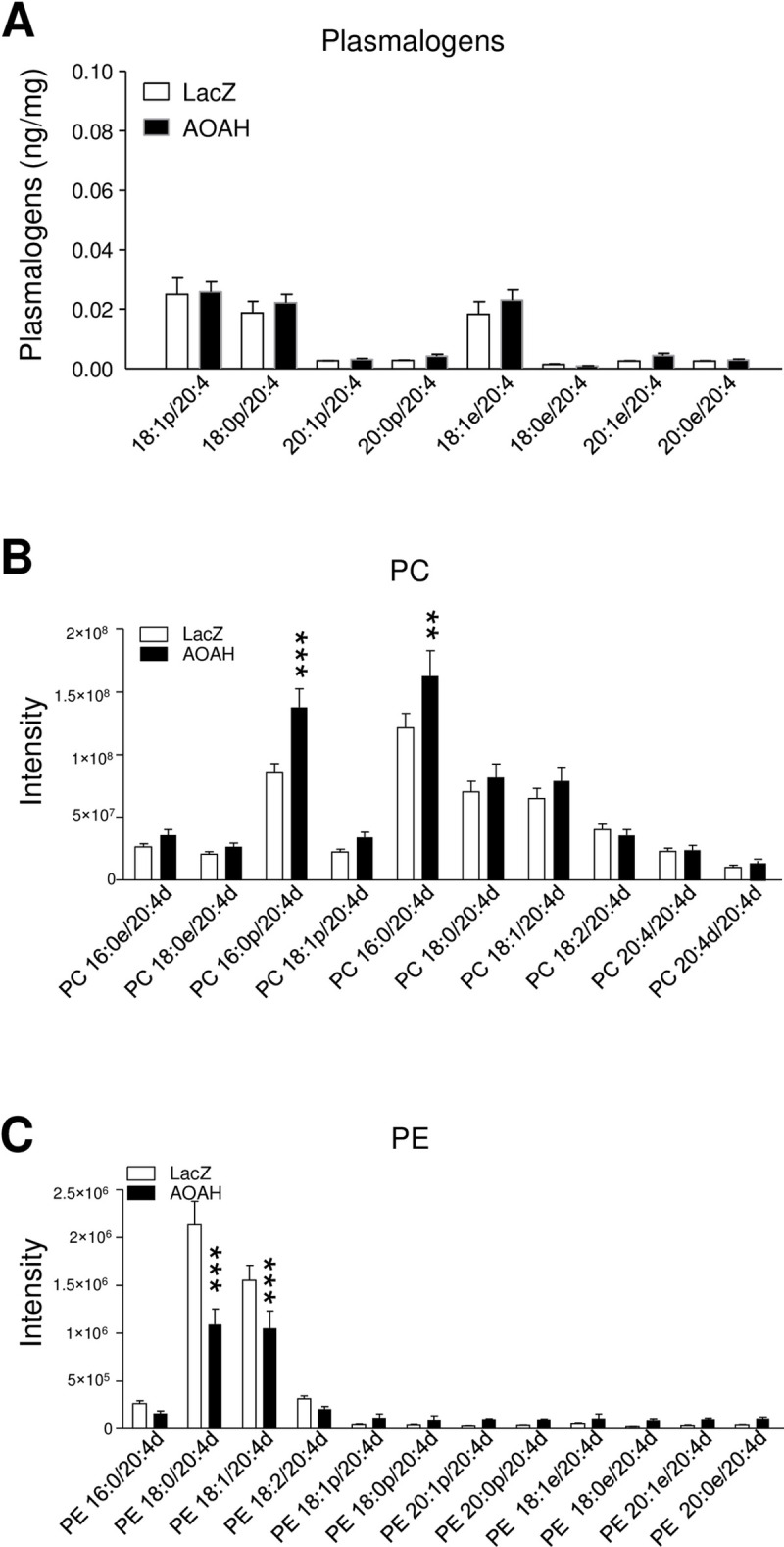
AOAH mediates AA incorporation into phospholipids. ***A*,** AA-containing plasmalogens were not significantly different in N42 lenti-LacZ neurons and N42 lenti-hAOAH3 cells. ***B*** and ***C*,** N42 cultures were treated with d8-AA for 30 min, and membrane lipids were analyses by shotgun lipidomics. AOAH expression significantly increased incorporation of AA into species of PC and significantly decreased incorporation in PE species (n = 5, **P < 0.01, ***P < 0.001, 2-way ANOVA).

N42 cells are a model of hypothalamic neurons generated by immortalization of murine embryonic neurons by SV40 large T antigen (Cellutions Biosystems, Toronto, Canada). We and others have used N42 cells previously to study mechanisms of *Crf* regulation, a gene known to be responsive to AA and eicosanoids [27–29]. Here, we used CRISPR to generate *Aoah*-null N42 neurons, using a guide RNA designed to target Exons 9 and 10 common to all functional AOAH isoforms, and we then transduced the *Aoah*-null N42 neurons with AOAH and lacZ lentiviruses to examine the effects of AOAH on AA distribution among neuronal phospholipids. In contrast to 293 cells, AOAH increased AA-containing plasmalogens only modestly under steady-state conditions (Fig 3A). To confirm that AOAH modulation of AA metabolism was not limited to 293s, we next examined incorporation of deuterated AA into N42 phospholipids. After labeling cultures for 30 min., the labeled PC and PE incorporated by [^2^H] AA were analyzed by MRM negative scan at m/z 311 (Q3), distinguishing from endogenous AA at m/z 303. Among ten [^2^H] AA-containing PC species that were identified, hAOAH3 significantly enhanced AA incorporation into the two most abundant PCs, 16:0p/20:4 and 16:0/20:4 (Fig 3B). In contrast, albeit nearly 100-fold less abundant relative to PC, the two most abundant PEs showed significantly *less* AA incorporation in N42 neurons expressing AOAH3, 18:0/20:4 and 18:1/20:4 (Fig 3C). These findings are counter-intuitive effects on AA metabolic labeling in light of the results from AOAH-deficient spinal cords (Fig 1). However, studies of AA metabolic labeling of monocytes under the conditions employed here showed a similar pattern of AA incorporation in PC and PE [30]. Together, these data suggest that AOAH modulates AA incorporation and distribution among phospholipid pools.

### AOAH alters membrane fluidity

**Fig 4:**
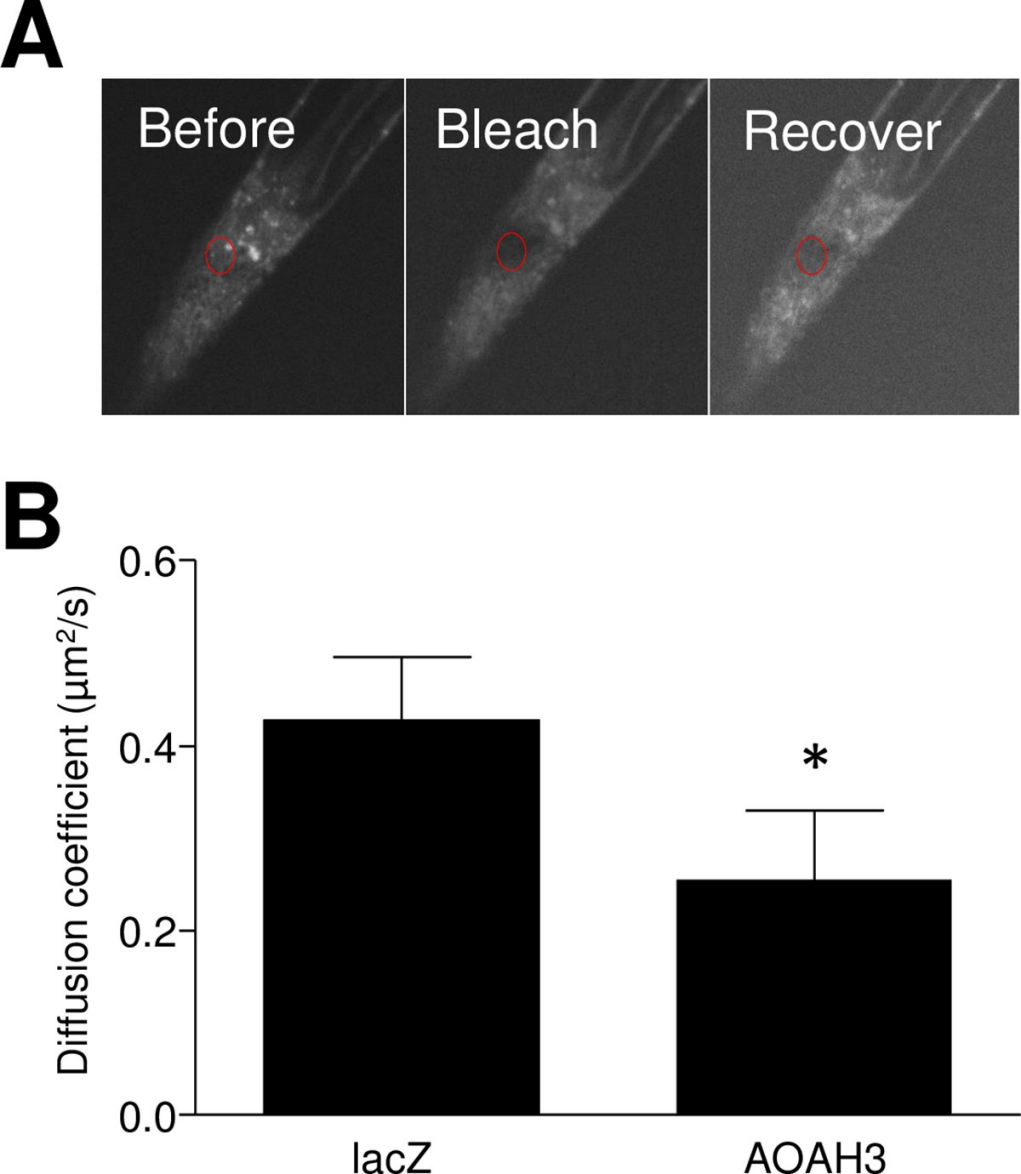
AOAH alters membrane fluidity. ***A***, FRAP image sequence displaying diffusion of BODIPY FL12 in the membrane of an N42 neuron. The circle indicates an area of photobleaching and subsequent recovery. ***B*,** N42 lenti-hAOAH3 neurons expressing AOAH3 have significantly reduced membrane fluidity, as detected by FRAP, relative to N42 lenti-LacZ cells expressing β-galactosidase (mean ± SEM, n = 15 and 7 cells respectively, *P < 0.05, Mann-Whitney).

Phospholipid composition can alter membrane properties. Because AOAH3 expression was associated with altered phospholipid composition, we next examined plasma membrane fluidity by fluorescence recovery after photobleaching (FRAP) [31]. Membranes of cultured N42 cells were labeled with the fluorescent fatty acid BODIPY FL-C12, a lipophilic dye that has been used previously for labelling membranes in a cyanobacterium [32]. The plasma membrane of individual cells was bleached by scanning within a circular area of 3 μm diameter at high laser intensity, and images were recorded during the recovery of fluorescence within the bleached area (Fig 4A). Diffusion coefficients were subsequently calculated using ImageJ with the SimFRAP plugin [25]. N42 cells expressing hAOAH3 recovered fluorescence more slowly than *lacZ* control cells, with diffusion coefficients of 0.255±0.074 SEM μm2/sec and 0.429±0. μm2/sec, respectively (Fig 4B, p = 0.048). These findings are consistent with a previous report that PE content modulates membrane fluidity [32].

### Suppression of pelvic pain of AOAH-deficient mice by EP-1 antagonist

**Fig 5:**
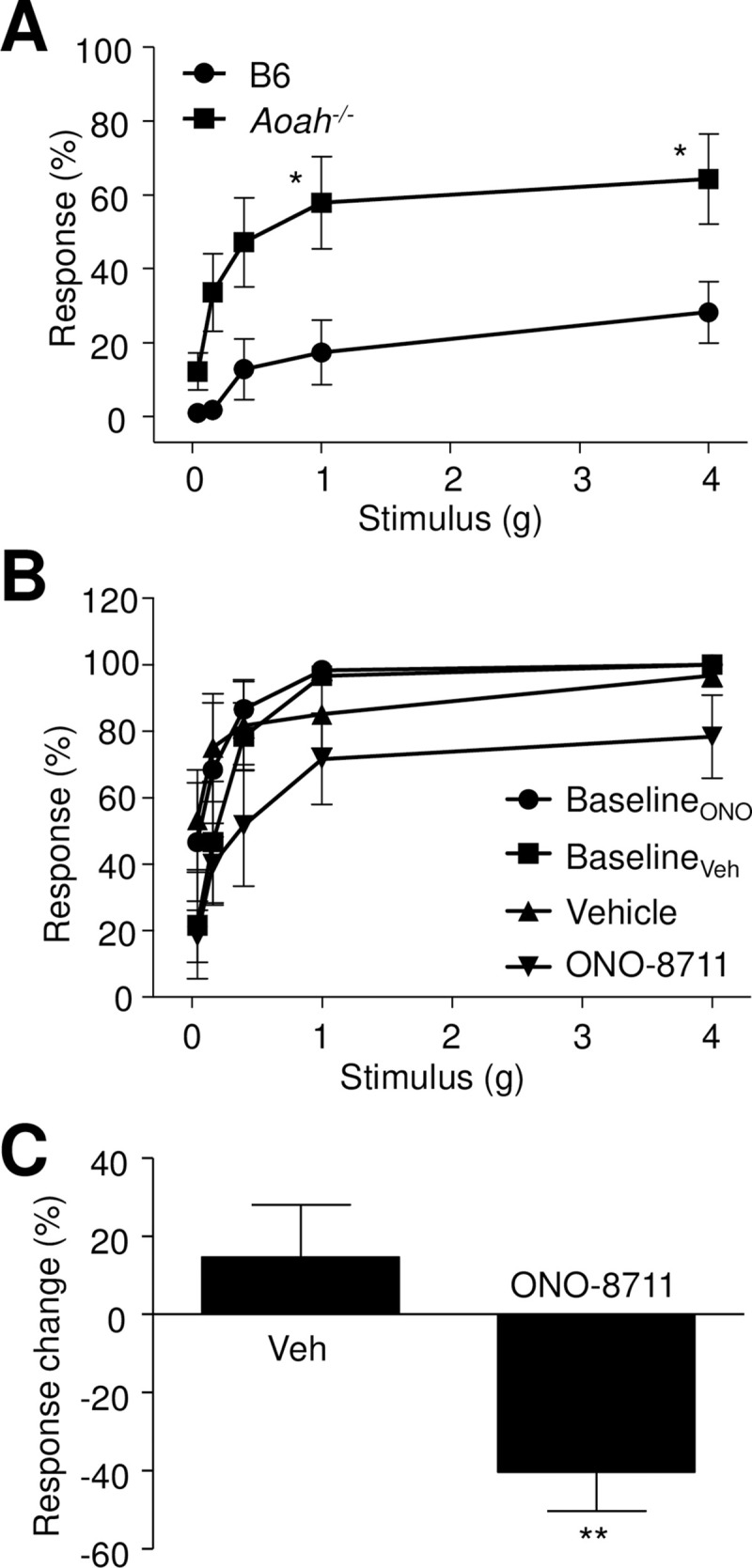
EP1 mediates pelvic pain. ***A***, Spontaneous pelvic allodynia is significantly higher in AOAH-deficient female mice than wild type mice in response to stimulation of the pelvic region with von Frey filaments (n = 10 mice, *P < 0.05 using 2-way ANOVA). ***B*** and ***C***, Sham-treated mice administered intrathecal saline (Vehicle) showed no significant change in allodynia relative to baseline (Baseline_Veh_). In contrast, mice treated with the EP1 antagonist ONO-8711 exhibited significantly decreased allodynia 1 h after treatment relative to baseline (Baseline_ONO_) (n = 5 mice, **P < 0.01, unpaired *t* test; see S1 File).

We previously reported that AOAH-deficient mice spontaneously develop pelvic mechanical allodynia consistent with referred pelvic pain and demonstrate here defects in AA homeostasis (Fig 1). PGE_2_ plays an important role in nociception and can drive sensitization in the spinal cord as well as in the periphery by acting through four PGE receptors, EP1, EP2, EP3 and EP4 [33]. Previous research showed that administration of an EP1 antagonist alleviated rat cystitis and modulated pain [34, 35]. To clarify the potential consequence of dysregulated AA homeostasis in the spontaneous pelvic pain phenotype of AOAH-deficient mice, we next examined the impact of EP1 antagonist ONO-8711 by quantifying pelvic mechanical allodynia in response to von Frey filaments applied to the pelvic region [5]. Briefly, 5 filaments of increasing stiffness (i.e., increasing applied force) were applied to the pelvic region ten times each and expresses at the percent responses to each stimulus force. As previously reported, female AOAH-deficient mice exhibited spontaneous pelvic pain as identified by pelvic tactile allodynia relative to wild type mice (Fig 5A). Next, AOAH-deficient mice were evaluated for response to the EP1 antagonist ONO-8711 in female AOAH-deficient mice. Intrathecal vehicle resulted in little change in allodynia for any stimulus intensity (i.e., applied force), whereas ONO-8711 injection resulted in reduced allodynia for each stimulus (Fig 5B). Overall, intrathecal administration of saline to female AOAH-deficient mice had no significant effect relative to baseline, where baseline sensitivity was determined as in Fig 5A and expressed as percent change across all 50 stimuli (10 applications of force with 5 fibers; Fig 5C). In contrast, i.t. treatment of female AOAH-deficient mice with ONO-8711 significantly decreased pelvic allodynia (Fig 5C). These data suggest that EP1 is a therapeutic target for treating spontaneous pain associated with AOAH deficiency.

## Discussion

Phospholipids comprise cellular membranes that provide barrier functions but also act as reservoirs of releasable fatty acids that are metabolized into bioactive lipids [16]. AA sequestration in phospholipids is a key step in modulating production of eicosanoids that cause pain and inflammation. AA is normally sequestered in membrane phospholipid pools and mobilized by phospholipase A2. For incorporation into phospholipids, AA is initially modified by covalent linkage to coenzyme A and then transferred to the free sn2 hydroxyl group of lysoPC to generate PC with AA at the sn2 position. AA is subsequently transferred to the sn2 hydroxl of lysoPE by a CoA-independent transferase to generate PE [16]. LysoPC is the major acyl acceptor for the initial incorporation of AA, then a CoA-independent transferase preferentially transfers AA moieties from diacyl-PC species to lysoPE species and is therefore responsible for maintaining the large amounts of ether-linked, AA-containing PE in tissues including the heart, brain, and leukocytes. Despite the central importance of an AA CoA-independent transferase to AA homeostasis, the protein(s) possessing CoA-independent transferase activity has not been identified in the nearly four decades since its first characterization [16]. The relative decrease of AA-containing PE species in sacral spinal cords of AOAH-deficient mice (Fig 1), combined with the relative increases in AA-containing PE species in cells over-expressing AOAH3 (Fig 2), suggests that AOAH possesses the capacity to transfer AA between phospholipids consistent with CoA-independent transferase activity. Thus, AOAH is a candidate AA CoA-independent transferase.

AOAH has the capacity to influence many biologic processes by virtue of mediating phospholipid homeostasis. Here we found that AOAH alters membrane fluidity (Fig 4), a finding that has implications for fundamental biological responses. In particular, neurons exhibit high membrane fluidity that varies with cellular development and likely influences neurite remodeling [36]. Moreover, AA incorporation is associated with synaptic vesicle formation and recycling (reviewed in [18]), thus suggesting a role for AOAH-mediated AA homeostasis in synaptic transmission. Membrane fluidity also influences the activity of ion channels and neurotransmitter receptors, both directly and indirectly by modulating the diffusion of regulatory factors such as G proteins (reviewed in [37]). Thus, AOAH-mediated effects on membrane fluidity have the potential to modulate nociception at multiple levels.

We find that AOAH-deficient mice have increased CNS AA and PGE_2_ [11]. Although the protein(s) encoding AA CoA-independent transferase activity has remained elusive, CoA-independent transferase inhibitors have been used extensively to probe the roles of transferases in AA incorporation and distribution among phospholipids (reviewed in [38]). For example, CoA-independent transferase inhibitors were employed to examine inflammatory responses in monocytes, and inhibiting CoA-independent transferase was found to block AA release and eicosanoid production [39]. These data are in contrast to our findings of increased AA/PGE_2_ in AOAH-deficient mice. However, treatment of lung carcinoma cell cultures with a CoA-independent transferase inhibitor enhanced accumulation of free AA [40], consistent with our own findings associated with AOAH deficiency in vivo. Together with our own findings, these studies suggest that AOAH mediates complex AA metabolism that may vary in a tissue-specific manner.

Prior studies noted that the amino terminal domain of AOAH resembles saposins, thus defining a saposin-like “SAPLIP” domain of AOAH that mediates lipid binding [41]. AOAH exists as a family of isoforms generated by alternative splicing, including the major isoforms AOAH1, 2, and 3. Several isoforms alter the degree to which the amino terminal SAPLIP domain is fully encoded, including a truncated SAPLIP domain in AOAH3. We speculate that AOAH isoforms alter substrate specificity for CoA-independent transferase reactions, perhaps even at a tissue- or cell-specific level. Likewise, *Aoah* transcripts also exhibit alternative splicing capable of altering the C-terminus that may provide additional modulation of CoIT activity. Thus, we speculate that AOAH could be involved in complicated lipid acyl transfer systems and AOAH’s activities could be extended further by alternatively spliced *Aoah* mRNAs [6].

These findings that AOAH mediates AA homeostasis provide mechanistic insights for AOAH in disease. Trans-eQTL studies found that *AOAH* expression was linked to numerous SNPs associated with chronic inflammatory diseases, such as rheumatoid arthritis and ulcerative colitis, suggesting a role in chronic inflammation [8]. Similarly, cardiovascular disease has an inflammatory component, and *AOAH* SNPs have been associated with carotid intima-media thickening, a marker of atherosclerosis [42]. Arachidonic acid metabolism plays a causal role in the development of atherosclerotic lesions through the actions of leukotrienes and prostaglandins in vascular change and macrophage activation (reviewed in [43]). Likewise, *AOAH* has been implicated in chronic rhinosinusitis [7, 9]. Chronic rhinosinusitis is classified into clinically distinct subtypes, including with/without nasal polyps and an association with aspirin exacerbated respiratory disease (reviewed in [44]), a condition of hypersensitivity to aspirin and other NSAIDS that immediately suggests links to AA metabolism. Chronic rhinosinusitis with polyps is typically characterized by an inflammatory infiltrate often consisting of eosinophils and other leukocytes known to release eicosanoids [45]. Although the complex pathophysiology is beyond the scope of this discussion and remains incompletely understood, chronic rhinosinusitis involves disruption of normal homeostasis provided by a balance of PGE_2_ signals (anti-inflammatory) and cysteinyl leukotrienes (pro-inflammatory), as well as altered expression of eicosanoid receptors on leukocytes and nasal epithelium. Thus, any alteration to AOAH-dependent AA homeostasis has the potential to alter normal eicosanoid balances and cell-cell interactions in chronic rhinosinusitis.

**Fig 6:**
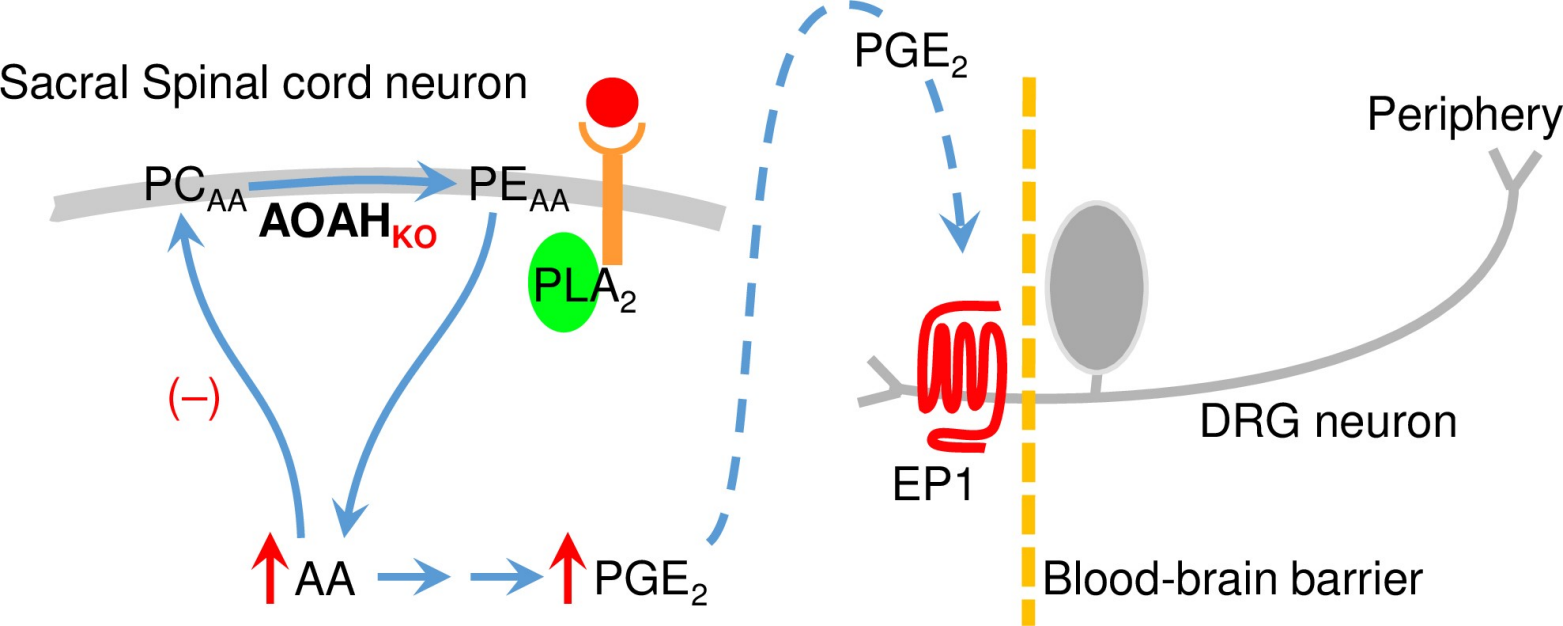
A model of AOAH function in AA sequestration and pelvic pain. Spinal cord AOAH CoIT activity mediates accumulation of AA in phosphatidyl ethanolamine. Reduced AOAH CoIT in AOAH deficiency results in reduced incorporation of AA in membrane (red “(–)”) and accumulation of free AA (red arrow) that is, in turn, metabolized and results in accumulation of elevated PGE_2_ (red arrow). Elevated PGE_2_ results in increased EP1 activation, sensitizing sensory neurons.

Likewise, AA homeostasis is critical to normal nervous system function, and disruptions may contribute to disease. The hypothalamic-pituitary-adrenal (HPA) axis is central to normal physiology, including stress responses [46]. The HPA axis is initiated by expression of corticotropin-releasing factor (CRF) in the hypothalamic paraventricular nucleus, and aberrant CRF signaling is associated with major depressive disorder [47–51]. *Crf* expression is responsive to eicosanoids [28, 52, 53], thereby establishing a link between stress responses and mood and AA metabolism. Indeed, we recently found that AOAH-deficient mice have increased CNS PGE_2_, increased *Crf* expression, HPA axis dysfunction, and depressive behaviors [11]. Finally, as reported here, we find AOAH-deficient mice exhibit evoked allodynia consistent with referred pelvic pain that is relieved by intrathecal administration of the EP1 antagonist ONO-8711 (Fig 5). *Aoah* was originally identified as a modulator of pelvic pain in a genetic screen using a mast cell-dependent pain model [5], raising possible mast cell involvement. However, to date RNAseq data have not reported expression of *Aoah* mRNA in human mast cells, and we have not found the antihistamine ranitidine reduces allodynia of AOAH-deficient mice, despite effectively reducing allodynia in a mast cell-dependent model pain [54, 55]. Together, these findings indicate that mast cells are not direct mediators of spontaneous and elevated pelvic pain in AOAH deficiency but rather may be transducers of altered efferent activity and/or trigger increased afferent activity. Instead, EP1 is expressed on dorsal root ganglion neurons and is known to enhance sensory input [56]. Together, these findings suggest a model where AOAH deficiency leads to elevated pain through increased production of PGE_2_ and subsequent sensitization of sensory inputs to pain circuits (Fig 6), a model supported by the efficacy of ONO-8711 in relieving pelvic mechanical allodynia. However, while accumulation of AA and PGE_2_ in AOAH-deficient mice and ONO-8711 efficacy are consistent with a neuron-centric model of AOAH in nociception, glia may also play a role. For example, microglia are known to modulate pain via several spinal mechanisms, including the release of PGE_2_ and pro-resolving lipid mediators that are regulated by sequestration of poly-unsaturated fatty acids in phospholipid pool [57]. Consistent with this potential mechanism, microglia have been found to express AOAH [58]. Thus, AOAH has the potential to alter normal processes and contribute to disease through altered AA metabolism in the periphery and the CNS.

In conclusion, our findings suggest that AOAH mediates AA homeostasis at the level of AA sequestration within phospholipid pools. Disruption of AA homeostasis normally provided by AOAH contributes to altered AA metabolism that may contribute to diverse pathophysiologies in the CNS and periphery.

## Supporting information

S1 File (PDF)

S1 Data (XLSX)

S2 Data (XLSX)

S3 Data (XLSX)

S4 Data (XLSX)
