# Supplementary material for: AOAH remodels arachidonic acid-containing phospholipid pools in a model of interstitial cystitis pain: A MAPP Network study
Source: PLoS One. 2020 Sep 14;15(9):e0235384. doi: 10.1371/journal.pone.0235384 (PMC7489500; doi:10.1371/journal.pone.0235384)
Supplement: S1 File — (PDF) [file pone.0235384.s001.pdf]

	Pre responses (of 50)	Post %responses (of 50)	% Pre	Increase
Veh expt 1	26	44	169.230769	69.2307692
	35	43	122.857143	22.8571429
	42	50	119.047619	19.047619
ono-8711 expt 1	27	9	33.3333333	-66.666667
	26	7	26.9230769	-73.076923
	42	50	119.047619	19.047619
ono-8711 expt 2	44	28	63.6363636	-36.363636
	43	37	86.0465116	-13.953488
	50	40	80	-20
	50	34	68	-32
Veh expt 2	27	29	107.407407	7.40740741
	26	18	69.2307692	-30.769231
	50	50	100	0
